# A novel ^18^F-labelled tetrazine ester prosthetic group for improved radiolabelling and in vivo stability of proteins and peptides

**DOI:** 10.1186/s41181-026-00430-6

**Published:** 2026-02-16

**Authors:** Francesco Lechi, Luke R. Odell, Olivia Wegrzyniak, Lorenzo J. I. Balestri, Olof Eriksson, Jonas Eriksson

**Affiliations:** 1 Science for Life Laboratory, Department of Medicinal Chemistry, Uppsala University, Dag Hammarskjölds Väg 14C, 3tr , SE-751 83 Uppsala, Sweden; 2 PET Center, Uppsala University Hospital, Uppsala, Sweden; 3 Department of Medicinal Chemistry, Uppsala University, 751 23 Uppsala, Sweden

**Keywords:** ^18^F-labelling, Tetrazine, IEDDA reaction, Biomolecule-PET

## Abstract

**Background:**

Monoclonal antibodies, engineered protein scaffolds, and peptides are increasingly important in therapy and diagnostics owing to their high specificity and affinity. Recent advances have enabled radiolabelling of such compounds with fluorine-18 in aqueous solution at room temperature via conjugation of [^18^F]tetrazines with trans-cyclooctene (TCO) moieties through the inverse electron-demand Diels–Alder (IEDDA) reaction. To simplify fluorine-18 labelling of large molecules, a novel tetrazine: 4-(6-methyl-1,2,4,5-tetrazin-3-yl)benzyl 6-[fluoro-^18^F]nicotinate ([^18^F]TzE2), was here developed and synthesized in a single-step procedure directly on a standard QMA cartridge used for trapping [^18^F]fluoride. The QMA containing [^18^F]fluoride was eluted over 2 min with *N*,*N*,*N*-trimethyl-5-(((4-(6-methyl-1,2,4,5-tetrazin-3-yl)benzyl)oxy)carbonyl)pyridin-2-aminium chloride in acetonitrile, forming [^18^F]TzE2 instantaneously as it passed through the cartridge. [^18^F]TzE2 was designed to combine the most favourable features of the previously described tetrazine prosthetic groups, [^18^F]TzAm and [^18^F]TzE, specifically, the superior in vivo performance and stability of [^18^F]TzAm, and the efficient and practical radiosynthesis of [^18^F]TzE. [^18^F]TzE2 was evaluated biologically as a reagent for direct labelling of the TCO-conjugated Affibody molecule Z09591, a high affinity marker for PDGFRβ.

**Results:**

The radiochemical yield for [^1^⁸F]TzE2 was 30.4 ± 3% (n = 4), corresponding to an activity yield of 5.8 GBq starting from 20 GBq [^1^⁸F]fluoride, with a total synthesis time of 13 min. [^18^F]TzE2 demonstrated greater stability in human plasma than the previous ester tetrazine [^18^F]TzE, but was significantly less stable than [^18^F]TzAm. The Affibody molecule Z09591 was successfully radiolabelled, giving an activity yield of 410 ± 160 MBq and > 90% radiochemical purity within a total synthesis time of 30 min from [^18^F]fluoride. [^18^F]TzE2-Z09591 exhibited improved plasma stability relative to [^18^F]TzE-Z09591, though lower than [^18^F]TzAm-Z09591. [^18^F]TzE2-Z09591 retained specific blockable binding to PDGFRβ-expressing human and murine tissues as demonstrated by in vitro autoradiography. Biodistribution of [^18^F]TzE2-Z09591 was rapid, with predominantly renal clearance, and showed improved targeting of PDGFRβ-expressing tissues compared with [^18^F]TzE-Z09591.

**Conclusion:**

The novel tetrazine prosthetic group [^18^F]TzE2 enabled straightforward radiolabelling of Affibody molecule Z09591 without affecting its binding properties. [^18^F]TzE2-Z09591 showed improved stability and in vivo targeting of PDGFRβ compared with the previously reported tetrazine ester [^18^F]TzE-Z09591. [^18^F]TzE2 represents a promising tetrazine prosthetic group for labelling of proteins and peptides with fluorine-18.

**Supplementary Information:**

The online version contains supplementary material available at 10.1186/s41181-026-00430-6.

## Introduction

Inverse electron-demand Diels–Alder (IEDDA) conjugation is widely used in bioconjugation and biomaterial chemistry due to its excellent biocompatibility, biorthogonal nature, catalyst-free process, rapid kinetics, and ability to be performed in aqueous media without heating. The reaction involves the cycloaddition between 1,2,4,5-tetrazines and strained alkene dienophiles, such as norbornenes and trans-cyclooctenes, to form a bicyclic compound with a dinitrogen bridge. This intermediate undergoes a retro-Diels–Alder reaction, releasing nitrogen gas to yield a stable dihydropyridazine product. The most common reaction pair in IEDDA chemistry are tetrazine and trans-cyclooctene (TCO) groups, which are also frequently employed in biomolecule radionuclide imaging and radiotherapy, where they serve as labelled prosthetic groups (Denk et al. 2014; Otaru et al. 2020; Morris et al. 2019; Oliveira et al. 2017; Zientek et al. 2023). In radiochemistry, the IEDDA click reaction has significantly impacted the synthesis and development of radiopharmaceuticals, particularly with fluorine-18. The technique has enabled the production of several ^18^F-labelled agents, such as the αvβ3-integrin-targeting peptide [^18^F]RGD, the GLP-1-targeting peptide [^18^F]exendin, and many others. The method’s compatibility with mild conditions and metal-catalyst-free nature make it suitable for sensitive biomolecules. Moreover, the IEDDA reaction is uniquely suited for pretargeted imaging probes, as it does not require additional heating (Reiner and Zeglis 2014).

**Fig. 1:**
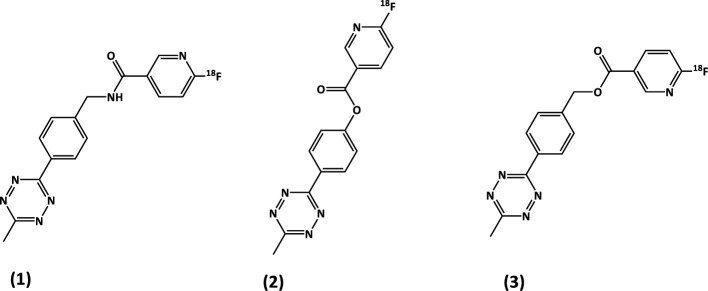
Structures of [^18^F]TzAm (**1**), [^18^F]TzE (**2**) and [^18^F]TzE2 (**3**), three compounds designed for IEDDA reaction with TCO-conjugated biomolecules

In this study, we present a comparison between three different ^18^F-labelled tetrazine prosthetic groups (Fig. 1), starting with two explored in previous projects: [^18^F]TzAm, an amide tetrazine (Fig. 1, compound **1**) (Denk et al. 2014; Olberg et al. 2010; Wegrzyniak et al. 2024), and [^18^F]TzE, an ester tetrazine (Fig. 1, compound **2**) (Lechi 2025; Munawar et al. 2024). [^18^F]TzAm was designed for IEDDA-based cycloaddition to TCO-conjugated biomolecules, and is produced via a solid-phase cartridge-based synthesis. Despite the efficacy of the cartridge-based method, which eliminates the need for azeotropic drying of [^18^F]fluoride (Richarz et al. 2014), the synthesis of [^18^F]TzAm is time-consuming, involving two reaction steps and requiring preparative HPLC purification (Zhou et al. 2019; Basuli et al. 2017, 2016; Syvänen et al. 2020). The Affibody molecule Z09591, which is a small 58 amino acid protein with high affinity for human and murine Platelet Derived Growth Factor Receptor β (PDGFRβ), demonstrated excellent receptor targeting and stability in vitro and in vivo. However, to further simplify the labelling protocol for large molecules, a second labelled tetrazine [^18^F]TzE was developed, which allowed facile one-step radiolabelling without HPLC purification. However, [^18^F]TzE-Z09591 demonstrated limited stability in human plasma, which negatively affected its performance as a PDGFRβ targeting PET tracer.

Therefore, a novel ester tetrazine, [^18^F]TzE2 (Fig. 1, compound **3**), was designed to reduce its sensitivity to esterases in circulation, to improve its stability both in plasma and in vivo. This modification involved replacing the phenol-derived ester with a benzylic derivative, which was anticipated to act as a less efficient leaving group, leading to enhanced stability.

The hypothesis was that [^18^F]TzE2 combines the favourable stability of [^18^F]TzAm with the synthetic simplicity of [^18^F]TzE, to generate a tetrazine-based prosthetic group for general radiolabelling of peptides and proteins via IEDDA cycloaddition. In this study, we performed in vitro and in vivo evaluations of [^18^F]TzE2-Z09591, in a direct comparison with [^18^F]TzAm-Z09591 and [^18^F]TzE-Z09591.

## Materials and methods

All reagents and solvents for synthesis and quality control (QC) were purchased from Merck (Darmstadt, Germany) or Thermo Fisher Scientific (Waltham, MA, USA). TCO-Z09591 precursor peptide was obtained via custom chemical synthesis (Affibody AB, Solna, Sweden). [^18^F]Fluoride was produced using a Scanditronix MC-17 (Scanditronix, Uppsala, Sweden) cyclotron by proton bombardment of oxygen-18-enriched water (enrichment grade 98%; Hyox Rotem Industries Ltd, Israel). The PS-HCO_3_^−^ cartridge, used as solid support for the ^18^F-labelling reaction, and the PS-H^+^-cartridge, used for intermediate purification, were purchased from Macherey–Nagel (Düren, Germany). The Sep-Pak tC18 cartridge used for reformulation was obtained from Waters (Milford, MA, USA). The custom-built Tracer Production System (TPS) at Uppsala University Hospital (Akademiska Sjukhuset, Uppsala, Sweden) was used for radiosyntheses, HPLC purification, and reformulation of the radiolabelled tetrazine compounds. Purification of the labelled Affibody molecules was performed using size-exclusion NAP-5 columns (Cytiva, Marlborough, MA, USA). The labelling precursors TzE2-Cl and TzE2-OTf were purchased from PharmaSynth AS (Tartu, Estonia).

### Synthesis of precursors for labelling of [^18^F]TzAm and [^18^F]TzE

**Scheme 1:**
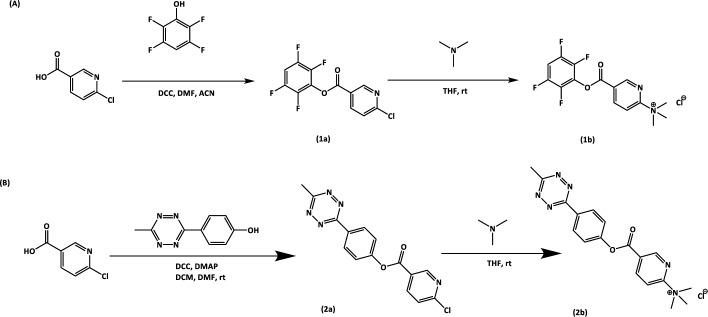
Synthesis of the [^18^F]TzAm (**A**) and [^18^F]TzE (**B**) precursors

The [^18^F]TzAm precursor (Scheme 1A) was synthesized by activation (esterification) of 3-chloro-6-nicotinic acid with 2,3,5,6-tetrafluorophenol (TFP), followed by formation of a quaternary ammonium salt through reaction with trimethylamine (TMA), in accordance with published procedures (Olberg et al. 2010). The [^18^F]TzE precursor was produced using a Steglich esterification as described elsewhere (Lechi 2025; Munawar et al. 2024) (Scheme 1B).

### Synthesis of [^18^F]TzAm and [^18^F]TzE

**Scheme 2:**
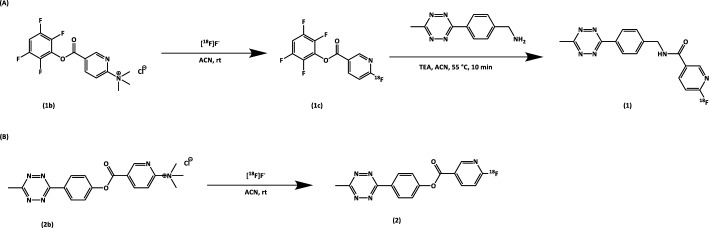
Synthesis of [^18^F]TzAm (**A**) and [^18^F]TzE (**B**)

All syntheses were performed with a starting activity in the range of 0.5–14 GBq. The synthesis of [^18^F]Tz-Am followed a two-step process (Scheme 2A) (Syvänen et al. 2020): cyclotron-produced [^18^F]fluoride was trapped on a QMA cartridge, rinsed with 5 mL of acetonitrile (ACN), and dried with air. A solution of precursor **1b** (10 mg) in acetonitrile (0.9 mL) was eluted at 500 µL/min, followed by acetonitrile (1.5 mL) and air, over the QMA into a reaction vial preloaded with (4-(6-methyl-1,2,4,5-tetrazin-3-yl)phenyl)methanamine (1 mg) in DMSO (100 µL). Next, 5 µL of triethylamine solution (10 µL TEA in 200 µL ACN) was added, and the mixture was heated at 55 °C for 10 min to produce **[**^**18**^**F]TzAm** (compound **1**).

Subsequently, 3 mL of water containing 0.1% TFA was added, the solution was mixed, and injected onto preparative HPLC (C18, 150 × 10 mm; Agilent 1260 Infinity II, Agilent Technologies, California, USA). The mobile phase consisted of solvent A = 0.1% TFA in water and solvent B = EtOH used for isocratic elution with 25% B and a flow rate of 0.7 mL/min. The retention time of compound **1** was 6.5 min (Supplementary Fig. S1).

The product-containing fraction was then collected and formulated in EtOH (0.5 mL) by solid-phase extraction (SPE), then evaporated under a nitrogen stream at 65 °C for 7 min.

[^18^F]TzE was synthesized in a single-step reaction on a QMA anion-exchange cartridge (Scheme 2B) (Lechi 2025). [^18^F]Fluoride was trapped on cartridge and rinsed with anhydrous ACN. A solution of precursor **2b** in ACN was then passed over the QMA cartridge, instantly forming [^18^F]TzE, and through a connected PS-H^+^ cation-exchange cartridge, which trapped the unreacted precursor. The cartridge assembly was subsequently flushed with ACN and air. The resulting solution of purified [^18^F]TzE was collected, diluted with an ascorbic acid solution in PBS, and reformulated in EtOH (0.5 mL) by solid-phase extraction using a tC18 Sep-Pak cartridge. The retention time of compound **2** was 2.7 min (Supplementary Fig. S2).

### Synthesis of [^18^F]TzE2

F-TzE2 reference compound (**3a**) and [^18^F]TzE2 precursor (**3b**) was synthesized as shown in Supplementary Scheme 1 and 2, respectively. Characterization of F-TzE2 reference **3a** is presented in Supplementary Figs. 3, 4. Characterization [^18^F]TzE2 precursor **3b** is presented in Supplementary Fig. S5.

**Scheme 3:**
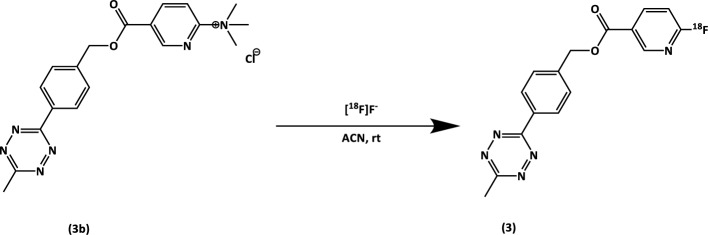
Synthesis of [^18^F]TzE2

[^18^F]TzE2 (**3**) was synthesized using a procedure analogous to that previously described for [^18^F]TzE (Scheme 3) (Lechi 2025). Target water (approx. 2 mL) containing cyclotron-produced [^18^F]fluoride (8.8–20 GBq) was trapped on an anion-exchange QMA cartridge, followed by a rinse with ACN (5 mL) and air, directed to a waste vial.

Next, 0.8 mL of precursor **3b** solution (1 mg, 2.5 µmol) in DMSO/ACN (800 µL, 1:15 v/v) was eluted at 800 µL/min over the cartridge. Subsequently, ACN (1 mL) was passed over the QMA cartridge. The precursor reacted with trapped [^18^F]fluoride, forming [^18^F]TzE2, which was readily solvated and eluted by the solvent. The solution continued through a PS-H^+^ cation-exchange cartridge into a 5 mL collection vial. Compared with the [^18^F]TzE protocol, the loading of the [^18^F]fluoride on the QMA cartridge was inverted, thereby shifting the exit zone of the cartridge so that, when [^18^F]TzE2 was formed, it had shorter distance to reach the cartridge outlet.

The collected [^18^F]TzE2 solution in ACN was diluted with PBS and reformulated by SPE using a tC18 cartridge, eluted with absolute EtOH (0.5 mL).

### Stability of [^18^F]TzE2 in acetonitrile

The stability of [^18^F]TzE2 in ACN was assessed by radio-HPLC analysis (Agilent 1290 Infinity II system, Agilent Technologies, California, USA) equipped with a Vydac 214MS C4 5 µm column (50 × 4.6 mm, Avantor, Pennsylvania, USA). The mobile phase consisted of solvent A = 0.1% TFA in water and solvent B = ACN, at a flow rate of 4 mL/min, with a linear gradient from 5 to 80% B over 10 min. The analysis was performed at three time points (0 min, 30 min and 90 min; n = 1 per timepoint). The retention time of [^18^F]TzE2 was 2.7 min (Supplementary Fig. S6).

### Radiolabelling of [^18^F]Tz-Affibody molecules

**Scheme 4:**
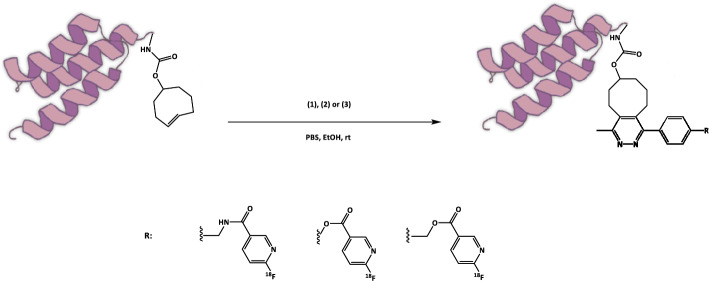
Radiolabelling of a Affibody molecule using ^18^F-labelled tetrazine

Three Affibody molecule conjugates, [^18^F]TzAm-Z09591, [^18^F]TzE-Z09591 and [^18^F]TzE2-Z09591 were radiolabelled via the IEDDA reaction with TCO-conjugated Z09591 at room temperature (Scheme 4).

For reactions with [^18^F]TzAm and [^18^F]TzE, the Affibody molecule TCO-Z09591 (100 µg, 14 nmol) was dissolved in PBS (300 µL), added to the tetrazine solution in EtOH, and allowed to react for 10 min.

For [^18^F]TzE2, TCO-Z09591 was dissolved in 500 µL of PBS and added to the solution of the radiolabelled tetrazine in EtOH (500 µL). An aliquot (500 µL) of the resulting 1:1 v/v PBS/EtOH mixture was collected and allowed to react for 10 min.

All three compounds were purified using NAP-5 size exclusion columns. The crude reaction mixture was loaded onto the column and eluted in three 500 µL fractions of 10% EtOH in PBS. The second fraction was collected and diluted with PBS for subsequent in vitro and in vivo evaluations.

### Radiochemical purity of [^18^F]Tz-Z09591

The radiochemical purity of the purified radiolabelled products was assessed by radio-HPLC. The analytical conditions were identical to those described for the stability assessment of [^18^F]TzE2.

Retention time was approximately 4.5–4.6 min for [^18^F]TzAm-Z09591 (Supplementary Fig. S7), [^18^F]TzE-Z09591 (Supplementary Fig. S8) and [^18^F]TzE2-Z09591 (Supplementary Fig. S9).

### In vitro stability in human plasma and in PBS formulation

Human plasma (anonymous donor samples from the local blood bank, obtained under ethical approval no. 2024–05050–01 from the Swedish Ethical Review Authority) was mixed with [^18^F]TzE2-Z09591, [^18^F]TzAm-Z09591 or [^18^F]TzE-Z09591 in PBS in a 6:1 plasma-to-compound solution volume ratio. The mixtures were incubated at 37 °C for 90 min (n = 3 per compound). After incubation, each sample was cooled on ice, and an equal volume of acetonitrile was added to precipitate plasma proteins. The resulting supernatant was filtered through a low protein binding 2 × 0.2 µm nylon membrane filters, and the filtrate was analysed by HPLC (up to 20 µl injection volume). The plasma stability assays and the HPLC protocol have been described previously (Wegrzyniak et al. 2024).

Separately, [^18^F]TzE2-Z09591, [^18^F]TzAm-Z09591 and [^18^F]TzE-Z09591 were incubated in PBS at room temperature (RT) for up to 60 min ([^18^F]TzE2-Z09591) and 90 min ([^18^F]TzAm-Z09591 and [^18^F]TzE-Z09591) to assess aqueous stability.

### In vitro autoradiography binding assay

An in vitro autoradiography binding assay was developed to investigate how the different tetrazine-based radiolabelling strategies affected the binding properties of the Affibody molecule Z09591.

Frozen tissue Sections (20 µm) of PDGFRβ-expressing U87 cell xenografts were obtained post-mortem from immunodeficient Balb/c nu/nu mice used in previous studies (ethical approval no. 05444/2024). For [^18^F]TzE2-Z09591, binding was also assessed on frozen tissue Sections (20 µm) representing different fibrotic pathologies and PDGFRβ expression, including human liver with metabolic dysfunction-associated steatohepatitis (MASH) (ethical approval no. 2019–02790), mouse heart infarct (ethical approval no. 01434/2023), mouse syngeneic MC38 model with tumour stroma (ethical approval no. 01552/2022) and rats with bleomycin-induced lung fibrosis (ethical approval no. 09018/2020).

Tissue sections were thawed and incubated in 150 mL PBS + 1% Bovine Serum Albumin (BSA) buffer at RT for 10 min. Next, [^18^F]TzE2-Z09591, [^18^F]TzAm-Z09591 or [^18^F]TzE-Z09591 was added at a target concentration of 5 nM (corresponding to ≈ 5–10 MBq / 150 mL), and the tissues were incubated for 60 min at RT. Separate sections were pre-treated and co-incubated with 2 µM unlabelled Z09591 to block the available PDGFRβ binding sites.

After incubation, the sections were washed twice for 1 min in cold PBS containing 1% BSA, once for 1 min in cold PBS, and finally dipped in MilliQ water. The sections were air-dried for 10 min and then exposed against a phosphor imaging plate (Cytiva) for around 4–6 h. Reference drops of known radioactivity were included to enable quantification of the results. The plates were digitalized using a Typhoon Phosphorimager (Cytiva) and visualized and analysed by ImageJ. The binding of each tracer was quantified as percentage of total binding, to assess the amount of specific binding that could be blocked by unlabelled cys-Z09591.

### In vivo biodistribution in Balb/c wild type and Balb/c immunodeficient mice

All the performed procedures were in accordance with the ARRIVE guidelines for reporting animal research. The animal experiments were ethically reviewed, approved by the Animal Ethics Committee of the Swedish Animal Welfare Agency and carried out in accordance with the European Directive 2010/63/EEC and the institutional guidelines at Uppsala University (UFV 2007/724).

The biodistribution over time of each of [^18^F]TzE2-Z09591, [^18^F]TzAm-Z09591 and [^18^F]TzE-Z09591 was evaluated in Balb/c wild type mice (female, n = 15, 23.0 ± 1.5 g). A target dose of 1 MBq of each tracer (0.8 ± 0.2 MBq, 100 µL) was injected intravenously and flushed with saline. After set time points (5, 10, 30, 60 or 120 min) groups of animals were euthanized, and tissues were removed, weighed, and measured for radioactive uptake by a gamma counter (Wizard, PerkinElmer). Tissues included blood, liver, spleen, muscle, lungs, kidney, bone, gastrointestinal (GI) tract, urine (when possible) and tail (to identify mice with failed injection). Tissue uptake was expressed as percentage per injected dose per gram of tissue (%ID/g).

Based on the result of the biodistribution over time above, a second organ distribution study was performed in Balb/c nu/nu immunodeficient mice at single time point after injection. Groups of mice (female, n = 14, 18.9 ± 1.4 g, n = 6 for [^18^F]TzE2-Z09591 and n = 4 each for [^18^F]TzAm-Z09591 and [^18^F]TzE-Z09591) were injected intravenously with a target dose 1 MBq of tracer (0.4 ± 0.1 MBq, 100 µL) and euthanized after 60 min. Tissues were processed and analysed as described above.

## Results

### Radiolabelling of [^18^F]TzAm-Z09591 and [^18^F]TzE-Z09591

[^18^F]TzAm and [^18^F]TzE were successfully synthesized following previously established protocols. [^18^F]TzAm was purified by preparative-HPLC and [^18^F]TzE by passage over an anion-exchange cartridge. The radiochemical yield was > 15% and the radiochemical purity was > 99% for both compounds.

After SPE reformulation in EtOH/PBS, [^18^F]TzAm and [^18^F]TzE were used in IEDDA cycloaddition with TCO-Z09591 to form [^18^F]TzAm-Z09591 and [^18^F]TzE-Z09591 at room temperature over 10 min. NAP-5 size-exclusion purification of [^18^F]TzAm-Z09591 and [^18^F]TzE-Z09591 gave the labelled compounds ready for injection, formulated in 10% EtOH in PBS. Both compounds were produced with similar radioactivity yields of 280 ± 110 MBq (n = 4) sufficient for PET applications. The molar activity of [^18^F]TzAm-Z09591 was 28 GBq/µmol and 44 GBq/µmol, and [^18^F]TzE-Z09591was obtained with a molar activity of 10 and 27 GBq/µmol.

### Stability of [^18^F]TzE2 in acetonitrile

Stability assessment of [^18^F]TzE2 in ACN demonstrated that the benzylic ester tetrazine was highly stable in solution. After 90 min in ACN, the compound retained a radiochemical purity of approximately 98%.

### Optimization of the synthesis of [^18^F]TzE2-Z09591

The radiolabelling of TzE2 was evaluated using the chloride (TzE2-Cl) and triflate (TzE2-OTf) precursors. TzE2-OTf exhibited excellent solubility in ACN, however, no ^18^F-fluorinated product was obtained when labelling was attempted using the solid-phase method. Therefore, all subsequent experiments were performed using TzE2-Cl. To fully dissolve the precursor in ACN, vigorous agitation for at least 60 s was required. Increasing the precursor load over 1 mg was not feasible due to its poor solubility. Using the TzE2-Cl precursor, [^18^F]TzE2 was obtained in initial tests with a radiochemical yield of approximately 10%, starting from 4 to 8 GBq of [^18^F]fluoride. After click reaction with TCO-Z09591 and NAP-5 purification, [^18^F]TzE2-Z09591 was obtained with an activity yield of on average 300 MBq and a radiochemical purity of > 99%.

The click reaction was evaluated in both EtOH and ACN, but reached completion only in EtOH, as expected (Lechi 2025; Syvänen et al. 2020). To improve the solubility of TzE2-Cl, various ratios of DMSO to ACN were evaluated (1:31, 1:15, 1:7, 1:4, 1:3 v:v). The best result was obtained at a 1:15 DMSO:ACN ratio, which improved the solubility of TzE2-Cl and increased the radiochemical yield of [^18^F]TzE2 by approximately 5%. The loading direction of [^18^F]fluoride on the QMA cartridge was inverted so that it was trapped near the cartridge outlet, thereby shifting the elution front of the TzE2-Cl solution and shortening the distance the formed [^18^F]TzE2 had to travel before exiting the cartridge. This configuration minimized the residence time of the formed product, avoiding excessive irreversible trapping on the solid-phase material. After solid-phase extraction reformulation, [^18^F]TzE2 was obtained in 500 µL EtOH. Performing the click reaction with TCO-Z09591 in neat EtOH produced negligible amounts of labelled product. It was determined that the click reaction tolerated a maximum EtOH concentration of 50% for full conversion. Hence, under optimized conditions, the click reaction was performed in a 1:1 (v:v) EtOH/PBS mixture, achieved by dilution with PBS to a total volume of 1 mL.

The click reaction was carried out with approximately 2.5 GBq of [^18^F]TzE2 by taking a 500 µL aliquot from the 1 mL [^18^F]TzE2 solution. The full volume was not used because it would overload the NAP-5 column during the subsequent purification step. The Affibody molecule TCO-Z09591 has a molecular weight of 7.032 kDa, and 100 µg was used in the labelling reaction. As the TCO-Z09591 was monofunctionalised, this corresponded to a maximum of 14 nmol of available TCO groups. The 2.5 GBq of [^1^⁸F]TzE2 corresponded to approximately 25 nmol of [^18^F]TzE2. Thus, theoretically [^18^F]TzE2 remained in excess relative to the available TCO groups, which may explain why the activity yield of [^18^F]TzE2-Z09591 did not increase with higher amounts of [^18^F]TzE2 starting activity.

The amount of TCO-Z09591 was the limiting reagent, so the activity yield did not improve even when significantly higher amounts of ^18^F-labeled tetrazine were applied.

Using the final optimized protocol, [^18^F]TzE2 was synthesized from 8.8 to 19.9 GBq of [^18^F]fluoride with a radiochemical yield of 30 ± 3% (n = 4) and an activity yield of 4.9 ± 1.9 GBq. The resulting [^18^F]TzE2-Z09591 was produced with an activity yield of 302 ± 171 MBq and a molar activity of 21 ± 14 GBq/µmol (n = 4).

### In vitro stability in human plasma and PBS

**Fig. 2:**
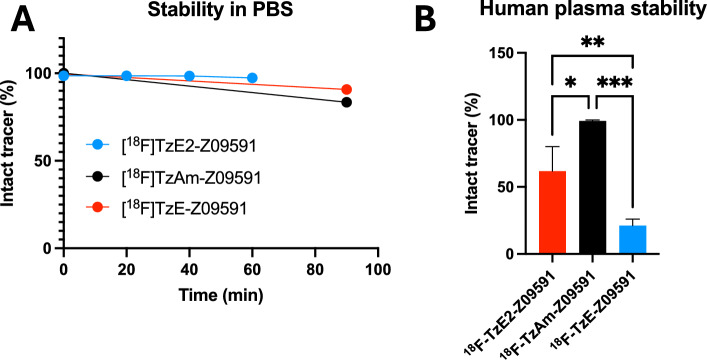
Stability of [^18^F]TzE2-Z09591, [^18^F]TzAm-Z09591 and [^18^F]TzE-Z09591 in PBS formulation over time (shelflife) (**A**) and after 90 min incubation in human plasma (**B**). One star (*) indicates *p* < 0.05, two stars (**) indicate *p* < 0.01 and three stars (***) indicate *p* < 0.001 using ANOVA for comparison between groups

[^18^F]TzE2-Z09591 was stable when formulated in PBS, maintaining a radiochemical purity of approximately 97% after 60 min, similar to [^18^F]TzAm-Z09591 and [^18^F]TzE-Z09591 (Fig. 2A). [^18^F]TzAm-Z09591 was highly stable in human plasma after 90 min of incubation, consistent with previous reports (99.1 ± 0.9%, n = 3). [^18^F]TzE2-Z09591 exhibited significantly higher stability than [^18^F]TzE-Z09591 (61.8 ± 18.3% vs 21.2 ± 4.8%, n = 3, p < 0.01, one-way ANOVA) (Fig. 2B).

### In vitro autoradiography binding study

**Fig. 3:**
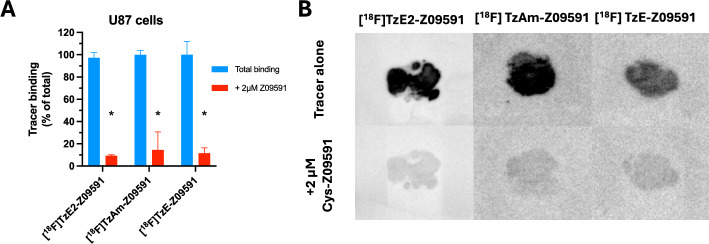
Binding of [^18^F]TzE2-Z09591, [^18^F]TzAm-Z09591 and [^18^F]TzE-Z09591 to frozen sections of PDGFRβ overexpressing U87 xenografts by an in vitro autoradiography assay. Quantification of binding signal as percentage of total binding (**A**). Representative autoradiograms for each of the tracers, both total binding (5 nM, top row panels), and after pre-blocking with 2 µM unlabeled Z09591 (bottom row panels) (**B**)

Z09591 radiolabelled with [^18^F]TzE2 retained excellent binding to PDGFRβ-overexpressing U87 tumour cells (Fig. 3A–B). The binding was abolished by approximately 90% by pre-blocking with excess unlabelled Z09591, demonstrating high binding specificity. The specificity was comparable to that of [^18^F]TzAm-Z09591 and [^18^F]TzE-Z09591, indicating that conjugation with the different tetrazine analogues did not negatively affect the binding affinity of Z09591. [^18^F]TzE2-Z09591 also demonstrated blockable binding to murine and human tissue sections representing various fibrotic pathologies including liver, heart, tumour stroma and lung (Supplementary Fig. S10A–D).

### In vivo biodistribution in Balb/c wild type and Balb/c immunodeficient mice

**Fig. 4:**
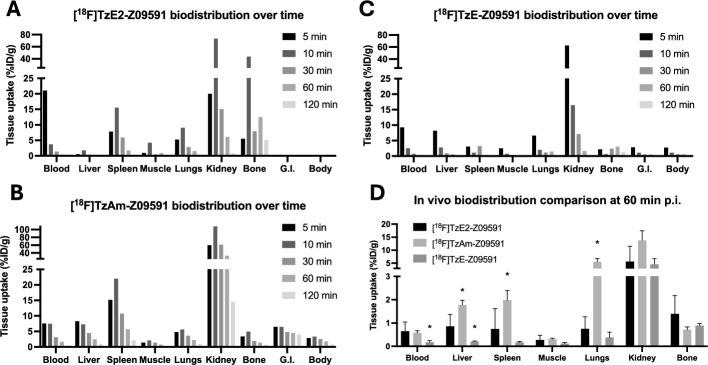
In vivo biodistribution over time of [^18^F]TzE2-Z09591 (**A**), [^18^F]TzAm-Z09591 (**B**) and [^18^F]TzE-Z09591 (**C**) in female wt Balb/c mice. Biodistribution was assessed by post-mortem organ distribution measurement by gamma counter, for up to 120 min after intravenous administration of each tracer. A direct comparison with more repeats (n = 4–6) in immunodeficient nu/nu Balb/c mice was compared at 60 min post-injection (**D**). A star (*) indicates difference compared to uptake of [^18^F]TzE2-Z09591

[^18^F]TzE2-Z09591 demonstrated rapid biodistribution and predominantly renal/urinary clearance in wild type (wt) Balb/c mice (Fig. 4A). A high initial blood signal was observed 5 min post-injection, which declined to near background levels by 60 min due to tissue uptake and excretion. Strong uptake was seen in the spleen, a positive control tissue with high PDGFRβ expression in mice (Supplementary Fig. S11), which disappeared after 120 min. Minimal uptake was detected in PDGFRβ-negative tissues, such as muscle. Uptake in the liver and gastrointestinal (GI) tract was low throughout the investigated time period, indicating negligible hepatobiliary excretion. Slight bone uptake was observed, potentially indicating minor incorporation of free [^18^F]fluoride.

In contrast, [^18^F]TzAm-Z09591 demonstrated slower clearance, predominantly renal but with partial hepatic excretion, and higher binding in PDGFRβ-positive spleen (Fig. 4B). [^18^F]TzE-Z09591, on the other hand, displayed very rapid urinary clearance, and minimal binding to spleen (Fig. 4C).

In a direct comparison performed in immunodeficient Balb/c nu/nu mice, 60 min post-injection, [^18^F]TzAm-Z09591 showed higher binding than [^18^F]TzE2-Z09591 in liver, spleen and lungs, indicating a longer circulation time (Fig. 4D). [^18^F]TzE-Z09591 instead showed lower uptake than [^18^F]TzE2-Z09591 in blood and liver.

## Discussion

The synthesis of tetrazine-based prosthetic group [^18^F]TzAm was effective, reproducible and consistently high-yielding, as was the subsequent conjugation via click chemistry to TCO-Z09591 (Wegrzyniak et al. 2023). However, the synthesis of [^18^F]TzAm required two reaction steps and an intermediate HPLC purification. The latter presented a clear disadvantage compared with the more practical one-step synthesis of [^18^F]TzE, which required no HPLC purification. To reduce the complexity of the radiolabelling protocol while combining the favourable features of [^18^F]TzAm and [^18^F]TzE, namely the high chemical stability and excellent in vivo properties of the former and the rapid one-step radiolabelling of the latter, a new tetrazine compound, [^18^F]TzE2, was designed as a suitable candidate (Fig. 1, compound **3**). The rationale behind the structural modification was that insertion of a –CH_2_– group would enhance the ester stability, reducing susceptibility to enzymatic cleavage (e.g., by esterases) in vivo.

**Scheme 5:**
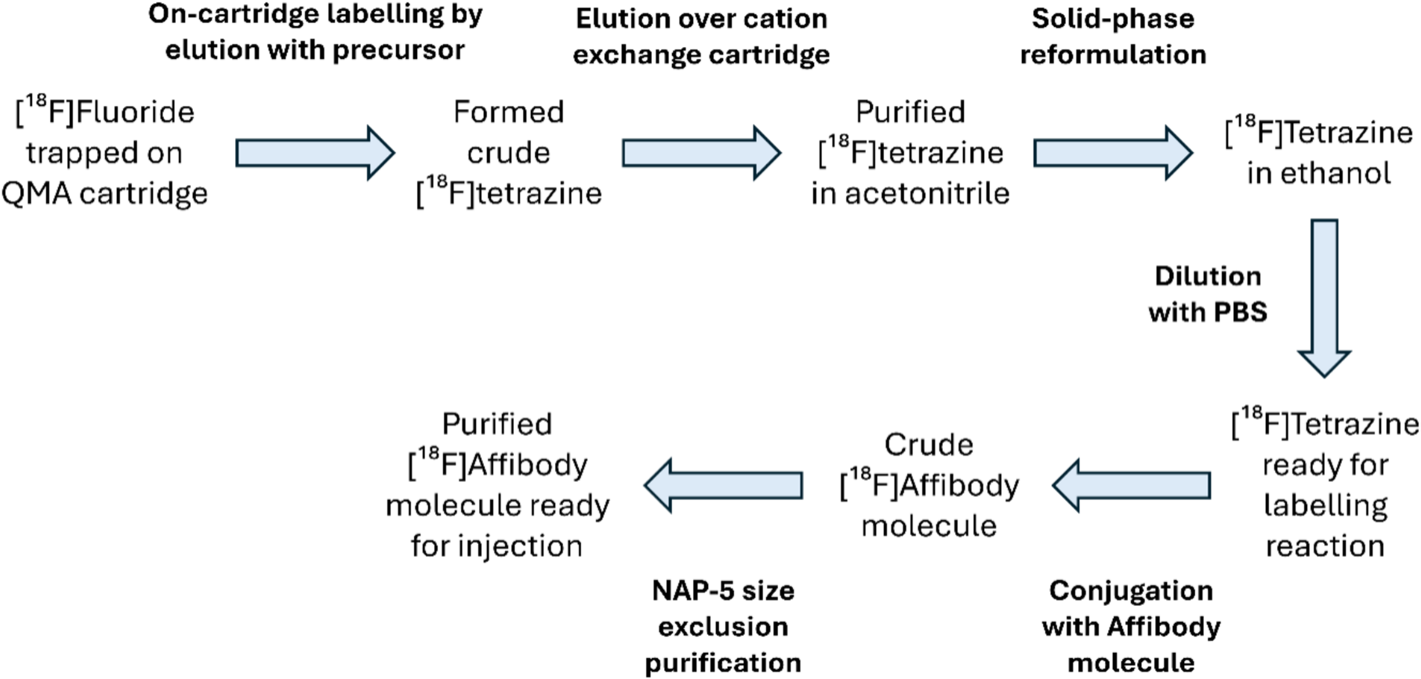
Workflow for ^18^F-radiolabelling of a TCO functionalised Affibody molecule via IEDDA conjugation of a [^18^F]tetrazine

During development of the labelling protocol, Scheme 5, two TzE2 precursors with identical molecular structures but different counterions (TzE2-Cl and TzE2-OTf) were evaluated. Unexpectedly, the highly soluble triflate precursor did not undergo successful labelling. In contrast, the chloride precursor (TzE2-Cl), though less soluble and therefore requiring modified solvent composition and vigorous agitation for preparation of the reaction solution, was successfully used to label [^18^F]TzE2. The tetrazine was subsequently used to radiolabel the Affibody molecule Z09591, which was site specifically functionalized with a single TCO-group.

The reason for the failed incorporation of the ^18^F-label in the case of the triflate precursor remains elusive. One hypothesis is that the high solubility of TzE2-OTf in ACN, may have limited its interaction with the solid support, thereby hindering nucleophilic substitution with [^18^F]fluoride trapped on the solid phase. In contrast, TzE2-Cl may undergo greater ion dissociation, increasing the availability of the reactive cation and may also be more apt to interact with the solid support leading to higher reactivity. Notably, adding DMSO to the precursor solution at a 1:15 (v/v) ratio improved the radiochemical yield, possibly by reducing the loss of precursor material due to precipitation prior to the start of the experiment and before reaching the reactive sites on the solid support.

The click reaction was tested both in EtOH and ACN but proceeded efficiently only in EtOH. This finding is consistent with previous reports (Lechi 2025; Syvänen et al. 2020), as ACN is generally less favourable for the inverse-electron-demand Diels–Alder (IEDDA) reaction.

To evaluate the labelling efficiency and in vitro stability of compounds labelled with [^18^F]TzE2, the Affibody molecule Z09591 was used as a model compound.

[^18^F]TzE2-Z09591 was successfully labelled with consistent radiochemical yields and high radiochemical purity. [^18^F]TzE2-Z09591 retained strong blockable binding to PDGFRβ-expressing tissues, including xenograft sections and multiple disease models, e.g. fibrotic pathology including PDGFRβ protein overexpression, including human fibrotic liver and murine heart infarcts, lung fibrosis and functional tumour stroma, indicating the potential versatility of this tracer across several indications. These results clearly indicate that the [^18^F]TzE2 radiolabelling strategy did not impair the binding capacity of the Z09591 Affibody molecule.

[^18^F]TzE2-Z09591 demonstrated excellent stability in PBS and markedly improved stability in human plasma (> 60% intact after 90 min) compared with [^18^F]TzE-Z09591 (< 25% intact). However, its stability remained lower than that of [^18^F]TzAm-Z09591. The combination of high stability of [^18^F]TzE2 in ACN and PBS, with intermediate stability in human plasma indicates a degree of susceptibility of the ester moiety to plasma esterases.

The improved stability of [^18^F]TzE2-Z09591 was also reflected by its increased tracer retention in blood compared with [^18^F]TzE-Z09591. A tendency of increased retention was observed in kidney, spleen and lung, suggesting prolonged systemic circulation of the tracer. The uptake in spleen is particularly interesting due to the endogenous expression of PDGFRβ in mouse spleen, and thus probably represents receptor mediated binding of [^18^F]TzE2-Z09591. Binding of [^18^F]TzE2-Z09591 in spleen was lower than that of [^18^F]TzAm-Z09591, reflecting lower in vivo stability of [^18^F]TzE2-Z09591 rather than differences in binding affinity, as confirmed by the autoradiography assay.

The use of radiolabelled tetrazines continues to gain importance in bioorthogonal chemistry, particularly for pretargeted PET imaging applications. Although high in vitro stability is generally desirable for radiopharmaceuticals, a moderate in vivo stability may be advantageous in this specific context. A slightly less stable [^18^F]tetrazine may facilitate faster metabolic degradation and systemic clearance, thereby reducing nonspecific accumulation and improving target-to-background ratios. Therefore, optimizing the balance between sufficient click reactivity and controlled in vivo stability could be a key parameter in development of effective tetrazine-based pretargeting agents.

Taken together, in vitro and in vivo results indicate that [^18^F]TzE2-Z09591 exhibited improved stability and in vivo performance compared with [^18^F]TzE-Z09591, though not reaching the level of [^18^F]TzAm-Z09591. These findings support partial protection from enzymatic hydrolysis conferred by the –CH_2_– insertion in the [^18^F]TzE2 structure, consistent with the decreased leaving group reactivity and enhanced steric hindrance associated with the benzyl ester motif.

## Conclusions

A rapid and high-yielding method for producing the ^18^F-labeled tetrazine [^18^F]TzE2 directly on a QMA cartridge was developed, eliminating the need for conventional [^18^F]fluoride drying and HPLC purification. The one-step procedure significantly simplifies the synthesis compared with the previously reported method for [^18^F]TzAm. [^18^F]TzE2 showed excellent stability in PBS and significantly improved stability in human plasma compared with [^18^F]TzE, enabling radiolabelling of the Affibody molecule Z09591 on the nanomole scale while producing sufficient radioactivity yields for PET applications. In vivo evaluation of the tracer was in concordance with the in vitro data, with improved stability and tissue uptake compared with [^18^F]TzE. These features establish [^18^F]TzE2 as a promising tetrazine prosthetic group for labelling proteins and peptides with fluorine-18.

## Supplementary Information

Supplementary Material 1.

## Data Availability

The datasets used and/or analysed during the current study are available from the corresponding author upon reasonable request.
