# Supplementary material for: A novel 18F-labelled tetrazine ester prosthetic group for improved radiolabelling and in vivo stability of proteins and peptides
Source: EJNMMI Radiopharm Chem. 2026 Feb 16;11:14. doi: 10.1186/s41181-026-00430-6 (PMC12929760; doi:10.1186/s41181-026-00430-6)
Supplement: Supplementary file 1 — Supplementary Material 1. [file 41181_2026_430_MOESM1_ESM.docx]

**Supplementary Data**

**A novel ^18^F-labelled tetrazine ester prosthetic group for improved radiolabelling and in vivo stability of proteins and peptides**

Francesco Lechi *et al.*

**Supplementary Methods**

**Supplementary Scheme S1.** Synthesis of a [^18^F]TzE2 reference (**3a**) compound via Steglich esterification: (4-(6-methyl-1,2,4,5-tetrazin-3-yl)phenyl)methanol (20 mg, 0.01 mmol) and DCC (20 mg, 0.01 mmol) were dissolved in 2.5 mL of DCM and left in agitation in inert N_2_ atmosphere at room temperature. A solution of fluoronicotinic acid (15.5 mg, 0.11 mmol) in 0.5 mL of DMF was added to the mixture, followed by 1.5 mg of DMAP (0.01 mmol) in 0.5 mL of DCM. The reaction was left overnight and monitored through TLC and LC-MS. The following day, another 1.5 mg of DMAP and 15.5 mg of fluoronicotinic acid (in 1mL DCM) were added to the crude mixture. After work-up with NaHCO_3_ and drying using MgSO_4_, the crude was over silica gel flash chromatography using 10% EtOAc in pentane. The purified product was dried, dissolved in 500 µL of deuterated chloroform and analyzed via ^1^H NMR and ^13^C NMR. (Supplementary Figure S3).

^1^H NMR (400 MHz, Chloroform-*d*) *δ* 8.89 (dd, *J* = 2.5, 0.8 Hz, 1H), 8.61 – 8.53 (m, 2H), 8.39 (ddd, *J* = 8.6, 7.5, 2.4 Hz, 1H), 7.65 – 7.55 (m, 2H), 6.96 (ddd, *J* = 8.5, 2.9, 0.7 Hz, 1H), 5.43 (s, 2H), 3.04 (s, 3H). ^13^C NMR (101 MHz, Chloroform-*d*) *δ* 167.5, 166.0 (d, ^1^*J*_CF_ = 246.7 Hz, 164.0, 163.8, 150.6 (d, ^3^*J*_CF_ = 16.6 Hz), 142.7 (d, ^3^*J*_CF_ = 9.5 Hz), 139.9, 132.0, 128.9, 128.3, 124.2 (d, ^4^*J*_CF_ = 4.5 Hz), 109.7 (d, ^2^*J*_CF_ = 37.5 Hz), 66.6, 21.2. MS (ESI) calculated for C_18_H_15_FN_6_O_2_ [M+CH_3_CN]^+^: 366.1; Found: 366.3

**Supplementary Scheme S2.** Synthesis of precursor for labelling of [^18^F]TzE2 (**3b**)**:** 2,3,5,6-tetrafluorophenyl 6-chloronicotinate (**4**) (281.6 mg, 0.92 mmol) was dissolved in a solution of tetramethylamine in THF (29 mL, 1M), stirred under nitrogen overnight, and monitored by TLC and LC-MS (Scheme 1A). After evaporating the THF, the crude was dissolved in Et_2_O, vacuum-filtered, and washed with Et_2_O and cold DCM. The product, dried under vacuum, was an opaque white solid (**5**), assessed by proton NMR, and further oven-dried for another NMR analysis in deuterated acetone. 249.9 mg of product were isolated, with a 75% yield. Finally, *N,N,N*-trimethyl-5-((2,3,5,6-tetrafluorophenoxy)carbonyl)pyridin-2-aminium (**5**) (90 mg, 0.25 mmol) was reacted with (4-(6-methyl-1,2,4,5-tetrazin-3-yl)phenyl)methanol (50 mg, 0.25 mmol) and TEA (50 mg, 0.49 mmol) in 10 mL ACN, stirred overnight under nitrogen, and monitored by TLC and LC-MS. The crude mixture, after solvent evaporation and vacuum drying, was purified via preparative HPLC (Agilent 1260 Infinity II, Agilent Technologies, California, USA) on a C18 column (Gemini 5µm C18 110Å, 250x10 mm, Phenomenex, California, USA) with a mobile phase made of solvent A= ACN and solvent B= 0.05% formic acid in H_2_O. The method was isocratic, with a solvent composition of A: B = 40:60. The chromatogram revealed three peaks; NMR showed peak 2 contained the purified product. Approximately 1 mg of pure **3b** was obtained from 10 mg of crude, which was then freeze-dried.

^1^H NMR (400 MHz, Methanol-*d*_4_) *δ* 9.16 – 9.15 (m, 1H), 8.74 – 8.63 (m, 1H), 8.52 – 8.49 (m, 2H), 8.09 – 8.00 (m, 1H), 7.74 – 7.59 (m, 2H), 5.49 (s, 2H), 3.60 (s, 9H), 2.96 (s, 3H). ^13^C NMR (176 MHz, D_2_O) *δ* 167.4, 165.0 ,163.9, 159.0, 150.3, 142.5, 140.0, 131.5, 129.1, 128.3, 128.2, 114.7, 67.4, 55.1, 20.1. MS (ESI) calculated for C_19_H_21_N_6_O_2_ [M]^+^: 365.2; Found: 365.0.

**Supplementary Figures**


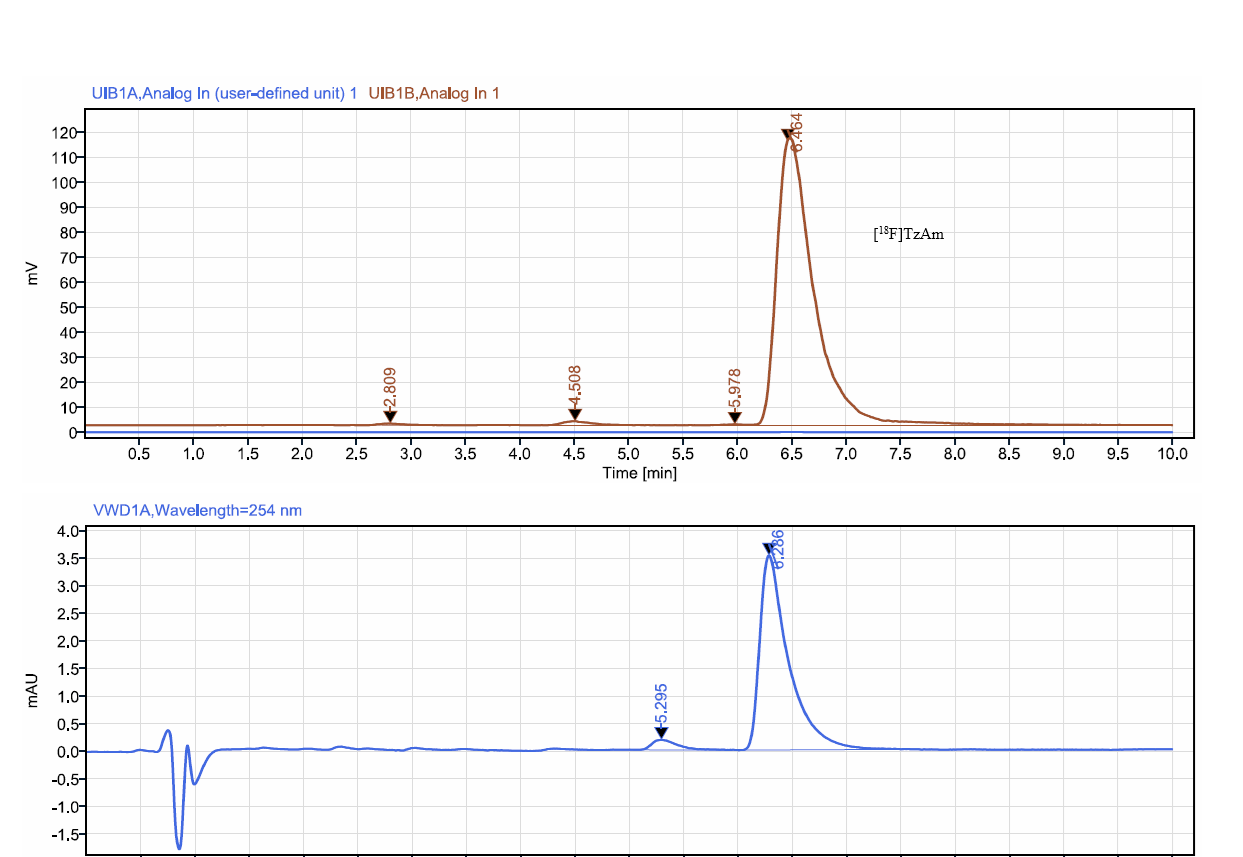


**Supplementary Figure S1. [^18^F]TzAm** Retention time: 6.5 min. HPLC column: Kinetex C18 2.6 μm 100 Å, 100 × 3.0 mm. Mobile phase: A = water and B = acetonitrile. Flow rate 0.7 mL/min. Isocratic elution 25% B over 10 min.


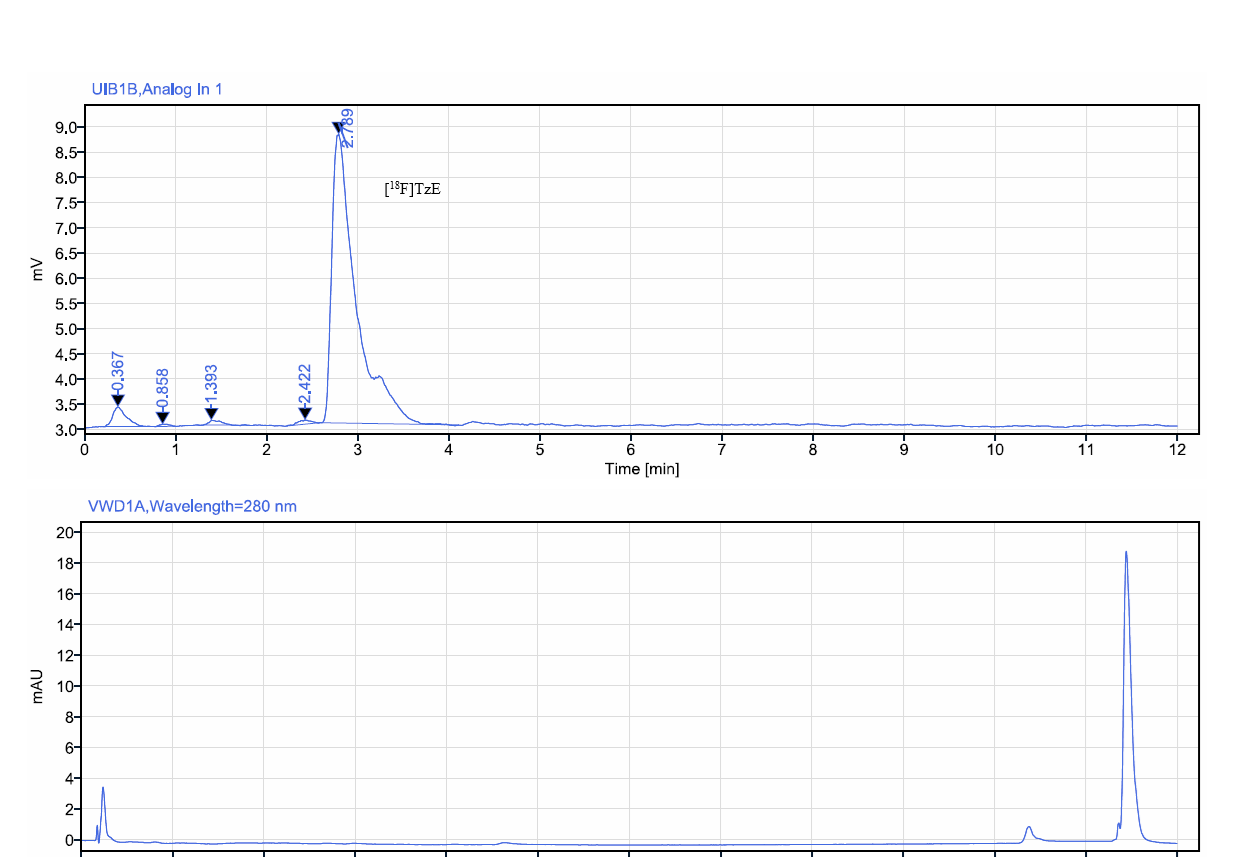


**Supplementary Figure S2. [^18^F]TzE** Retention time: 2.7 min. HPLC column: Vydac 214MS C4 5 µm 50 x 4.6 mm. Mobile phase: A = 0.1% TFA in water and B = acetonitrile. Flow rate 4 mL/min. Gradient: Linear from 5% to 80% B over 10 min.


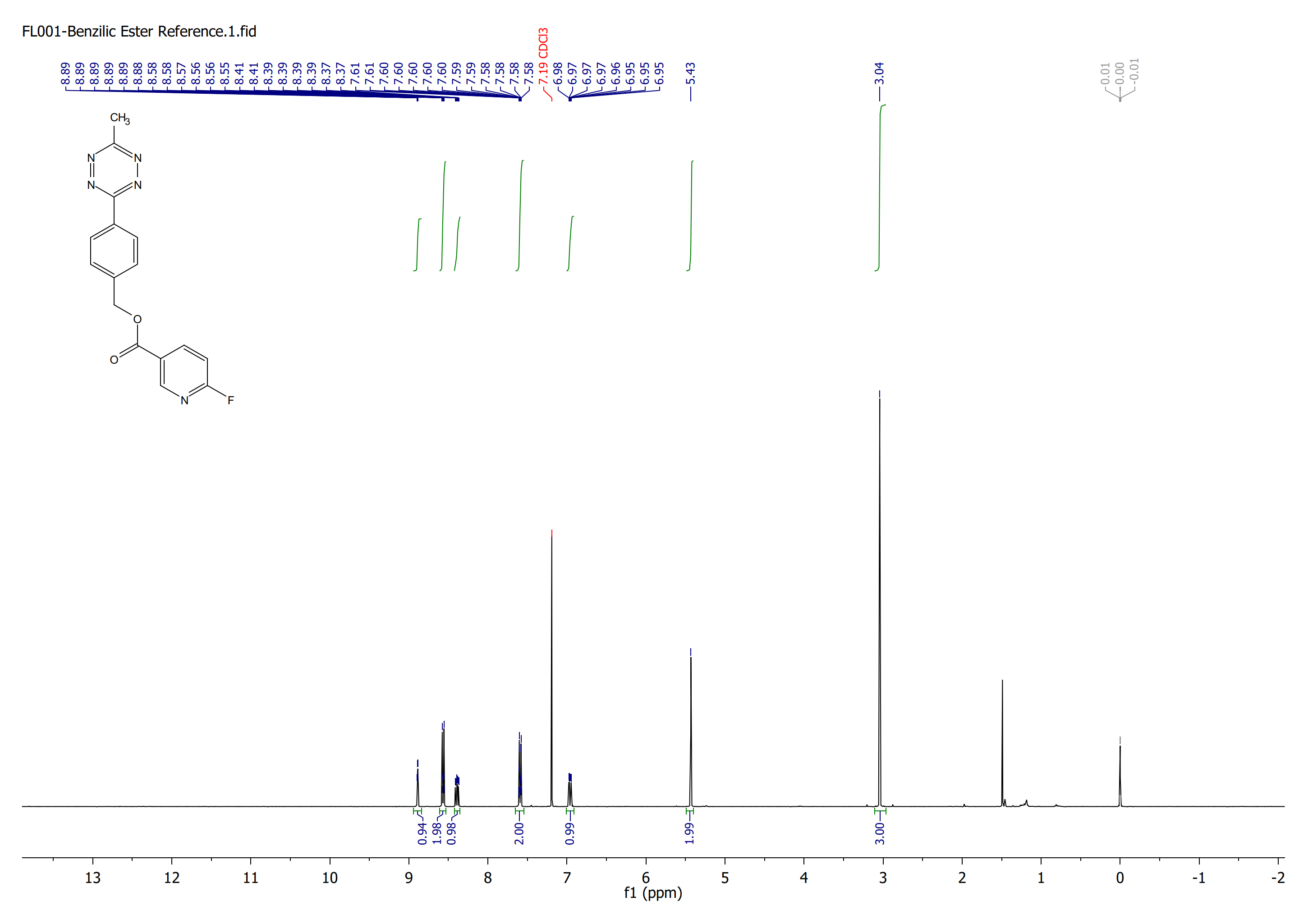


**Supplementary Figure S3.** ^1^H NMR spectrum of reference compound F-TzE2 (**3a**).

**Supplementary Figure S4.** ^13^C NMR spectrum of reference compound F-TzE2 (**3a**).

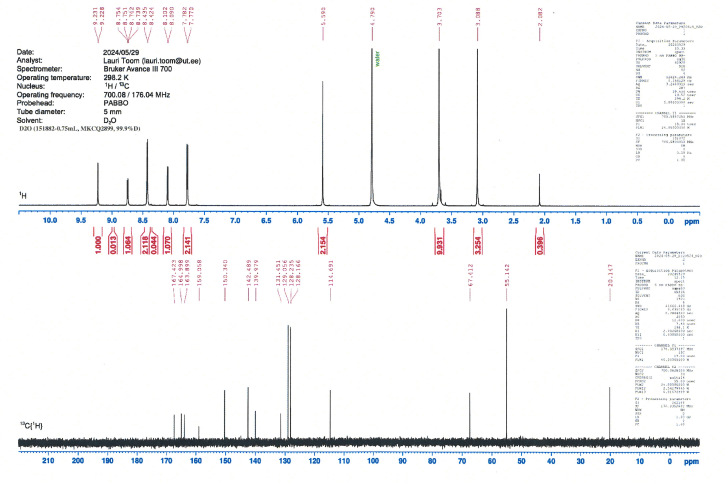


[Grab your reader’s attention with a great quote from the document or use this space to emphasize a key point. To place this text box anywhere on the page, just drag it.]

**Supplementary Figure S5.** ^1^H and ^13^C-NMR spectrum of precursor for labelling of [^18^F]TzE2 (**9**).


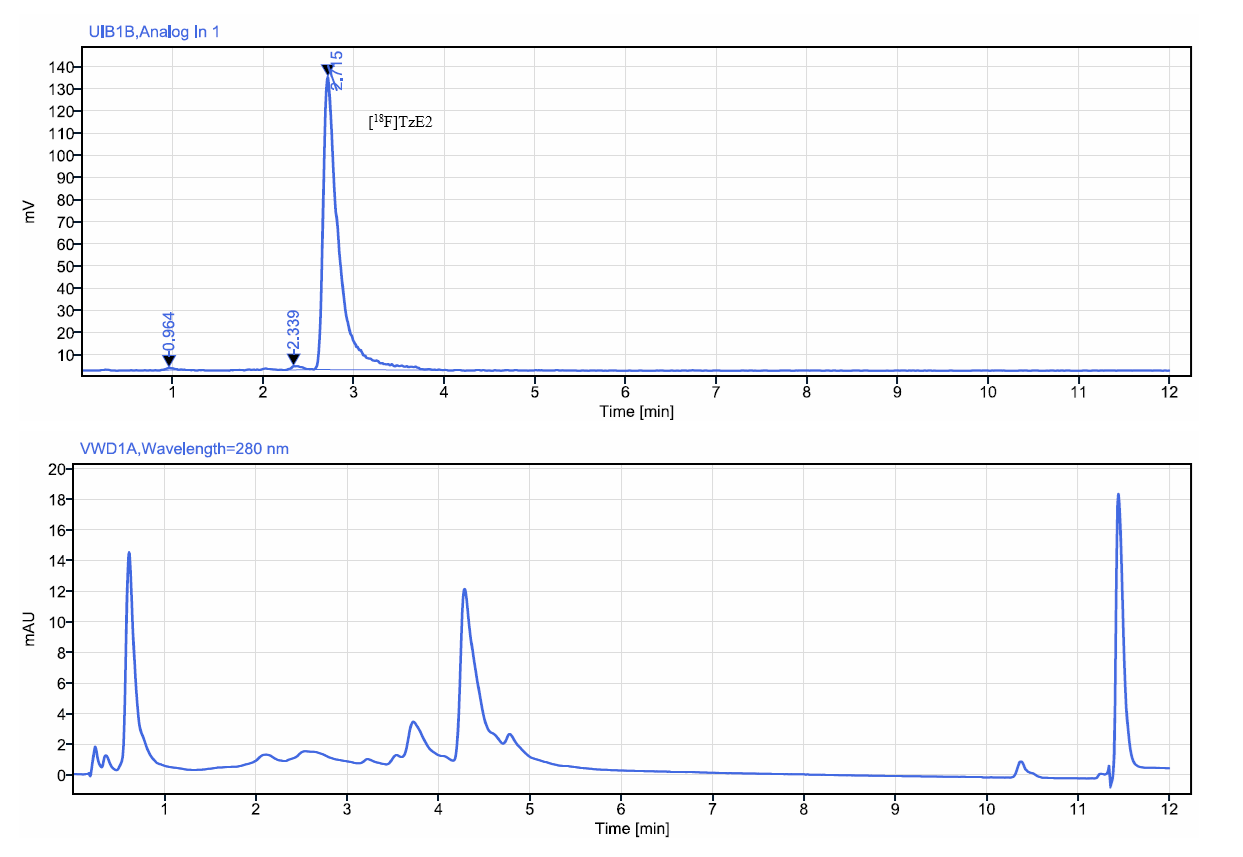
 **Supplementary Figure S6. [^18^F]TzE2** Retention time: 2.7 min. HPLC column: Vydac 214MS C4 5 µm 50 x 4.6 mm. Mobile phase: A = 0.1% TFA in water and B = acetonitrile. Flow rate 4 mL/min. Gradient: Linear from 5% to 80% B over 10 min.


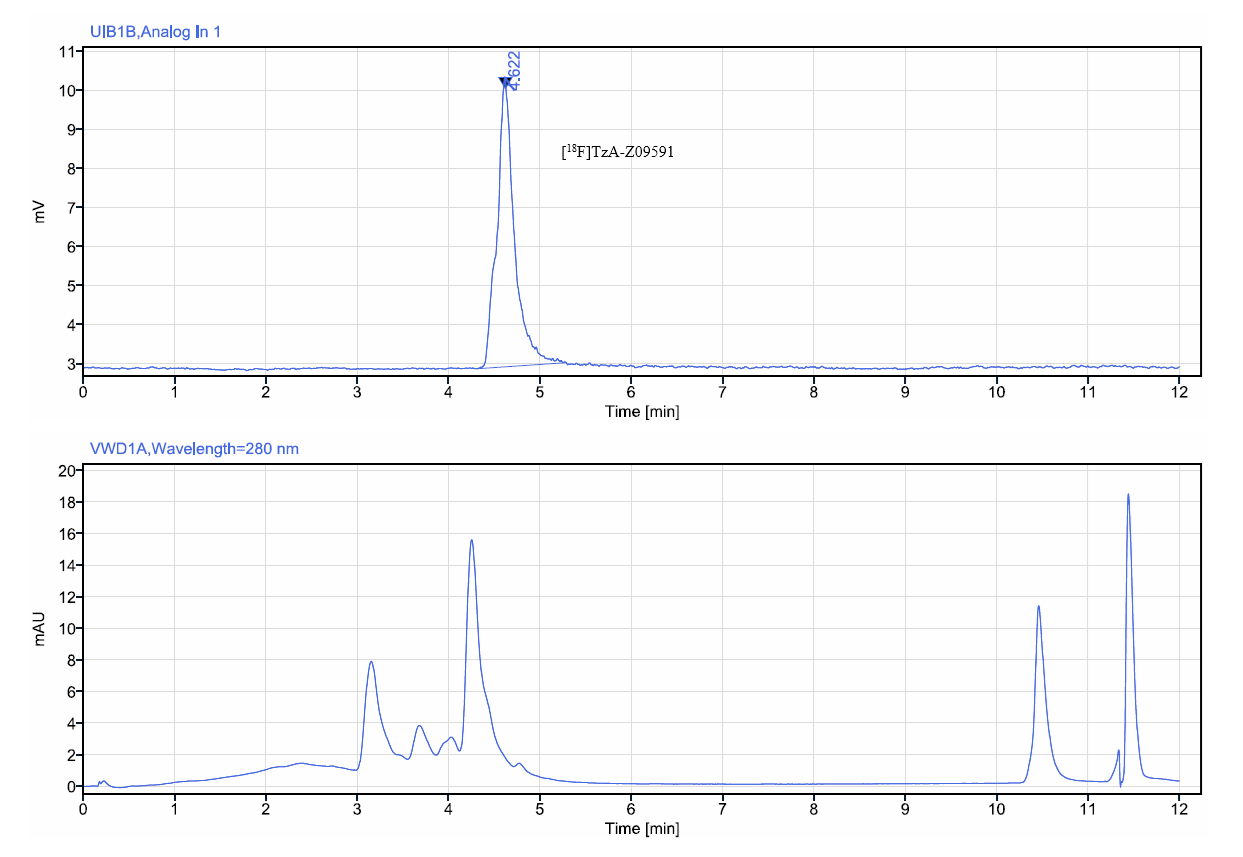


**Supplementary Figure S7. [^18^F]TzAm-Z09591** Retention time: 4.6 min. HPLC column: Vydac 214MS C4 5 µm 50 x 4.6 mm. Mobile phase: A = 0.1% TFA in water and B = acetonitrile. Flow rate 4 mL/min. Gradient: Linear from 5% to 80% B over 10 min.


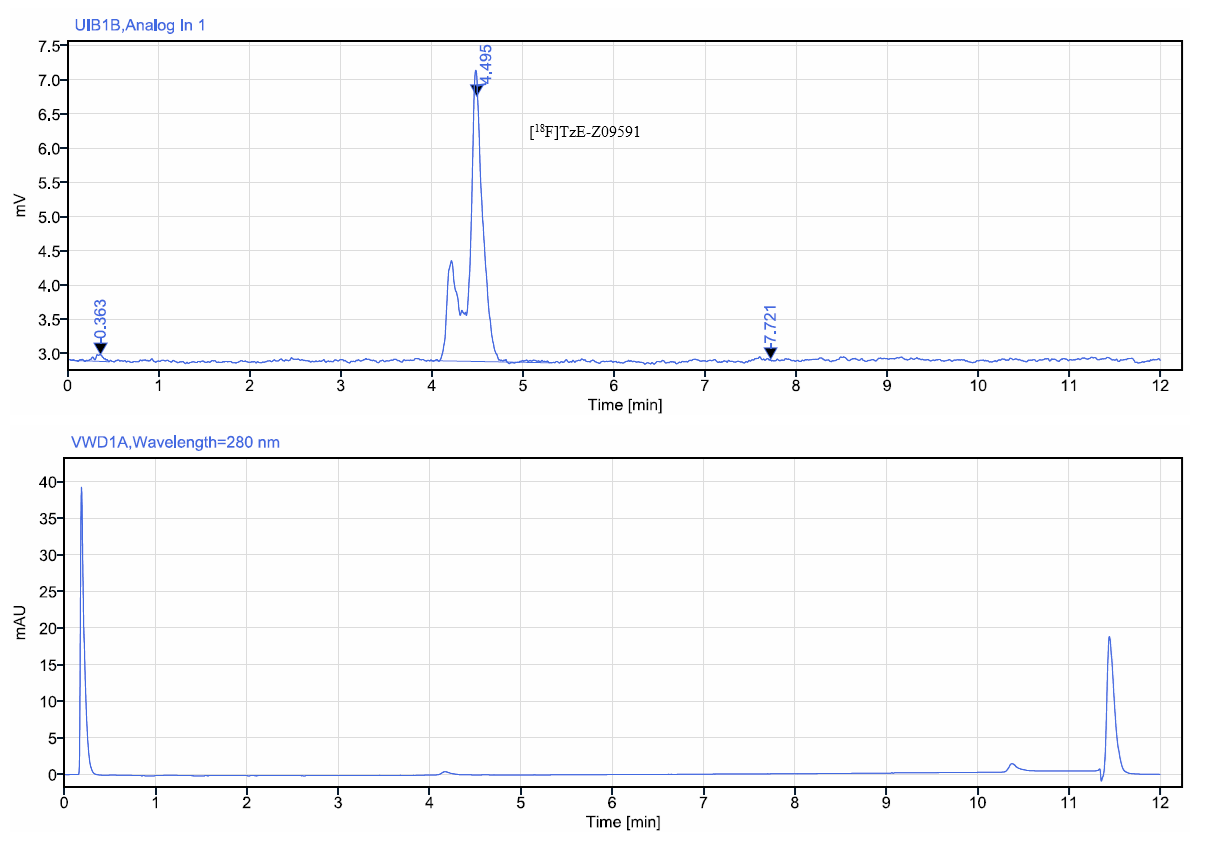


**Supplementary Figure S8. [^18^F]TzE-Z09591** Retention time: 4.5 min. HPLC column: Vydac 214MS C4 5 µm 50 x 4.6 mm. Mobile phase: A = 0.1% TFA in water and B = acetonitrile. Flow rate 4 mL/min. Gradient: Linear from 5% to 80% B over 10 min.


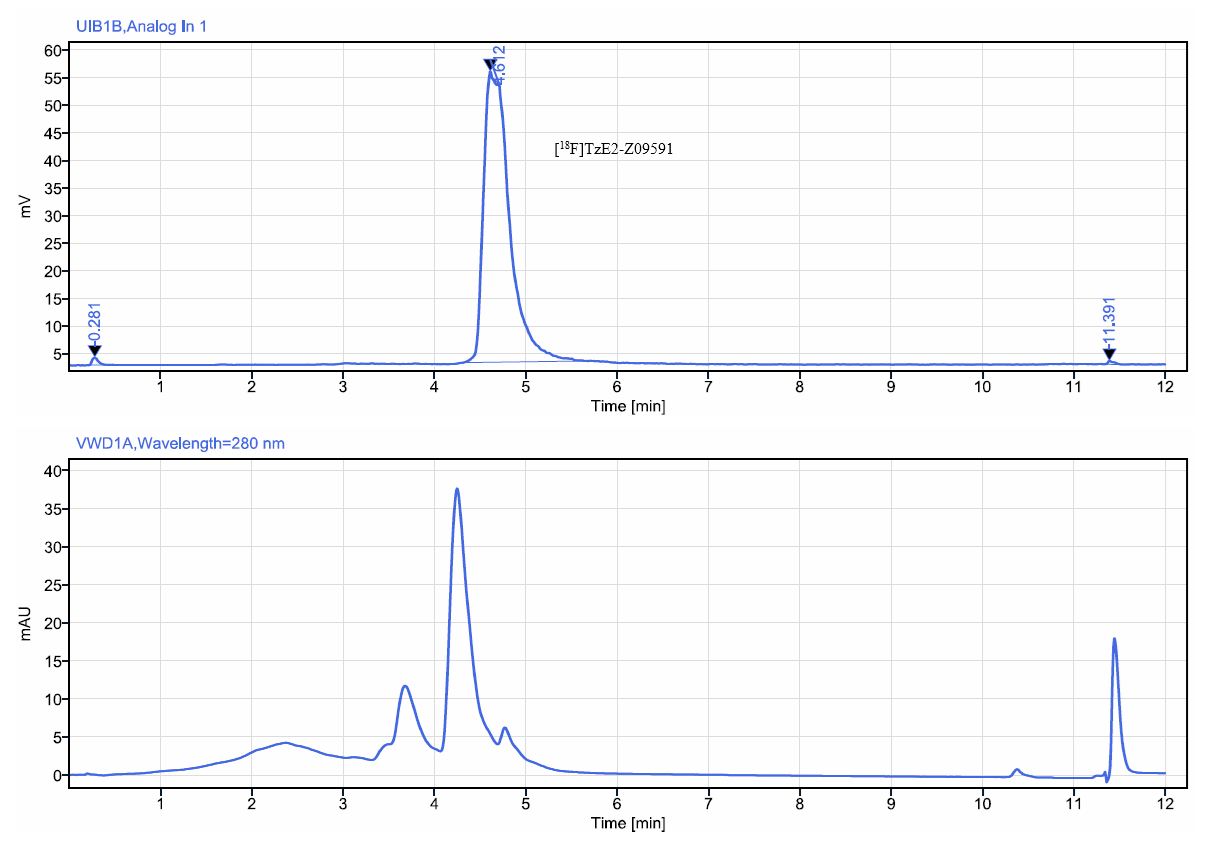


**Supplementary Figure S9. [^18^F]TzE2-Z09591** Retention time: 4.6 min. HPLC column: Vydac 214MS C4 5 µm 50 x 4.6 mm. Mobile phase: A = 0.1% TFA in water and B = acetonitrile. Flow rate 4 mL/min. Gradient: Linear from 5% to 80% B over 10 min.

**Supplementary Figure S10.** Representative autoradiograms showing binding of [^18^F]TzE2-Z09591 to several tissues with different kinds of fibrotic pathology, including human MASH liver (A), mouse heart with and without induced infarct (B), mouse spleen, muscle and a syngeneic MC38 colorectal tumor with functional tumor stroma (C) and rat spleen, muscle and bleomycin treated lung (D). Top rows show autoradiograms after incubation with 5 nM [^18^F]TzE2-Z09591, while the bottom row show sequential sections pre-blocked with 2 µM Cys-Z09591.

**Supplementary Figure S11.** Representative histological staining of tissues from a U87 xenografted immunodeficient mice, including muscle (“Mu”), spleen (“Sp”) and tumor. Left panel show staining for Masson’s Trichome (MTC), middle panel show immunostaining for PDGFRβ in brown and right panel show staining for Hematoxylin/Eosin (H/E).
